# Targeting Interleukin‐6 as a Novel Strategy to Overcome Eribulin Resistance in Breast Cancer

**DOI:** 10.1002/cam4.71273

**Published:** 2025-11-04

**Authors:** Akira Hattori, Masayuki Nagahashi, Miki Komatsu, Sayaka Urano, Mamiko Kuroiwa, Yosuke Matsushita, Toyomasa Katagiri, Masafumi Shimoda, Yasuo Miyoshi

**Affiliations:** ^1^ Division of Breast and Endocrine Surgery, Department of Surgery Hyogo Medical University Nishinomiya Hyogo Japan; ^2^ Laboratory of Biofunctional Molecular Medicine National Institutes of Biomedical Innovation, Health and Nutrition Osaka Japan

**Keywords:** breast cancer, drug resistance, eribulin, interleukin‐6, tocilizumab

## Abstract

**Background:**

In breast cancer, elevated serum interleukin‐6 (IL‐6) level is associated with a poor prognosis during eribulin treatment; however, the mechanisms underlying IL‐6‐mediated resistance and its potential as a therapeutic target remain unclear. We aimed to determine whether IL‐6 is involved in eribulin resistance and evaluate the therapeutic potential of combining eribulin with an IL‐6 receptor‐specific inhibitor to overcome resistance.

**Methods:**

Eribulin‐resistant cell lines (MCF‐7E and MDA‐MB‐231E) were established through prolonged culture with eribulin. IL‐6 in conditioned media was quantified using enzyme‐linked immunosorbent assay, and RNA‐seq was performed to identify pathways associated with eribulin resistance. The therapeutic efficacy of eribulin in combination with tocilizumab (TCZ), an IL‐6 receptor inhibitor, was assessed using a patient‐derived xenograft (PDX) mouse model with acquired eribulin resistance.

**Results:**

The IC_50_ of eribulin in both MCF‐7E and MDA‐MB‐231E cells was > 500‐fold higher than that of their parental counterparts. RNA‐seq revealed significant activation of several signaling pathways, particularly the IL‐6–JAK–STAT pathway, in eribulin‐resistant cells but not in doxorubicin‐resistant MCF‐7D cells. Gene set enrichment analysis confirmed the enrichment of IL‐6–JAK–STAT pathway‐related genes in MCF‐7E and MDA‐MB‐231E. Although TCZ alone had no effect on cell viability, its combination with eribulin had a synergistic inhibitory effect on MDA‐MB‐231 cells. In the PDX model, tumor growth in the eribulin + TCZ group was significantly suppressed compared with that in the eribulin‐only group.

**Conclusions:**

These findings suggest that IL‐6 contributes to eribulin resistance in breast cancer and that IL‐6 receptor inhibition is a promising therapeutic strategy to overcome this resistance.

**Abbreviations**
ALC: absolute lymphocyte count
BME: basement membrane extract
ERI: eribulin
GSEA: gene set enrichment analysis
MCF‐7D: doxorubicin‐resistant MCF‐7 cells
MCF‐7E: eribulin‐resistant MCF‐7 cells
MDA‐MB‐231E: eribulin‐resistant MDA‐MB‐231 cells
NLR: neutrophil‐to‐lymphocyte ratio
PDO: patient‐derived organoid
PDX: patient‐derived xenograft
TCZ: tocilizumab
WST‐8: water‐soluble tetrazolium salt 8

## 1 Introduction

Breast cancer remains the most prevalent malignancy among women worldwide, and despite substantial therapeutic advances, recurrence and metastasis continue to account for a substantial proportion of breast cancer–related mortality [1, 2, 3]. Systemic chemotherapy forms the cornerstone of treatment for metastatic breast cancer (MBC); however, repeated exposure to anticancer agents frequently leads to the development of drug resistance [4, 5, 6]. Eribulin, a non‐taxane microtubule dynamics inhibitor, has been shown to improve overall survival (OS) in patients with MBC, despite having limited effects on progression‐free survival (PFS) [7]. Notably, the phase III EMBRACE trial demonstrated that patients with a high baseline absolute lymphocyte count (ALC) experienced significantly prolonged OS following eribulin treatment compared to those who received physician's choice therapy [8]. Several subsequent studies have reported associations between eribulin efficacy and immune‐related parameters, including ALC and neutrophil‐to‐lymphocyte ratio (NLR), further implicating the host immune response in modulating treatment outcomes [9, 10, 11, 12, 13, 14, 15, 16]. Nevertheless, the mechanistic links between eribulin efficacy and immune modulation remain unclear. We recently reported that elevated serum interleukin‐6 (IL‐6) level in patients with breast cancer is associated with poor prognosis during eribulin therapy [17].

IL‐6 is a proinflammatory cytokine implicated in tumor progression and resistance to anticancer therapies [18, 19, 20]. In breast cancer, tumor‐derived IL‐6 exerts both autocrine and paracrine effects, promoting cancer cell survival and modulating the tumor immune microenvironment [21, 22]. Specifically, IL‐6 has been shown to support tumor progression by acting directly on cancer cells and by promoting immune tolerance through the expansion of myeloid‐derived suppressor cells (MDSCs) [17, 23, 24]. Although IL‐6 is known to drive cancer progression and therapeutic resistance, its role in mediating resistance to eribulin in breast cancer has not been previously investigated.

The aim of this study was to elucidate the contribution of IL‐6 to eribulin resistance using eribulin‐resistant breast cancer cell lines developed through long‐term exposure to eribulin. Additionally, we explored the potential of combining eribulin with an IL‐6 receptor‐specific inhibitor, tocilizumab (TCZ), as a strategy to overcome resistance and enhance therapeutic efficacy.

## 2 Materials and Methods

### 2.1 Cell Lines and Culture Conditions

The human breast cancer cell line MCF‐7 was obtained from EMD Millipore and maintained in phenol red–free DMEM/F12 medium (Sigma, cat. no. D6434) supplemented with 10% fetal bovine serum (FBS; EMD Millipore, cat. no. ES‐009‐B), 2.5 mM l‐glutamine (EMD Millipore, cat. no. TMS‐002‐C), 6 ng/mL insulin (Sigma, cat. no. I‐9278), and antibiotic–antimycotic solution (Gibco, cat. no. 15240096) [23]. MDA‐MB‐231 cells were cultured in Leibovitz's L‐15 medium (Gibco, cat. no. 11415‐064) supplemented with 10% FBS (BioWest, cat. no. S1810‐500) and antibiotic–antimycotic solution under atmospheric air conditions.

### 2.2 Establishment of Eribulin‐Resistant Cell Lines

Eribulin‐resistant cell lines were generated from MCF‐7 and MDA‐MB‐231 cells through prolonged exposure to increasing concentrations of eribulin (Eisai Co. Ltd., Tokyo, Japan) [23]. The resulting resistant cell lines were designated MCF‐7E and MDA‐MB‐231E, respectively. For MDA‐MB‐231 cells, treatment commenced with 0.3 nmol/L eribulin, with subsequent dose escalation by 1.5‐fold increments until a final concentration of 656 nmol/L was reached. MCF‐7 cells were initially treated with 1 nmol/L eribulin, and the concentration was gradually increased by a 1.5‐fold factor until a final concentration of 675 nmol/L was achieved. Doxorubicin‐resistant MCF‐7 cells (MCF‐7D) were established using an analogous protocol.

### 2.3 Cell Viability Assay

Drug sensitivity was assessed using Cell Count Reagent SF (WST‐8; Nacalai Tesque, cat. no. 07553‐44) according to the manufacturer's instructions. Briefly, 2000 cells/well were seeded in 96‐well plates and incubated in 90 μL of culture medium for 24 h. Subsequently, 10 μL of medium containing various concentrations of eribulin was added. After 96 h, culture supernatants were collected and stored at −80°C. Thereafter, 90 μL of Dulbecco's phosphate‐buffered saline (D‐PBS; Nacalai Tesque, cat. no. 14249‐24) mixed with 10 μL of WST‐8 reagent was added to each well. After 2 h of incubation, absorbance was measured at 450 nm using a SPECTRAmax PLUS384 microplate reader (Molecular Devices, CA, USA). To evaluate combinatorial effects, impedance‐based cell viability measurements were performed using CytoView‐Z 96‐well plates and the Maestro‐Z system (Axion Biosystems). The plates were precoated with 1 μg/mL fibronectin (Sigma) for 1 h at room temperature and seeded with 10,000 cells/well. TCZ, a clinically approved humanized monoclonal antibody that inhibits the IL‐6 receptor, was selected for this study because its clinical efficacy has been confirmed in patients with multiple diseases, including rheumatoid arthritis [25]. After 24 h, the cells were treated with eribulin, with or without TCZ, and impedance was recorded for 48 h. All experiments were performed in triplicate.

### 2.4 RNA Sequencing and Pathway Analysis

Total RNA was extracted using the NucleoSpin RNA kit (Takara Bio) following the manufacturer's protocol. RNA purity and integrity were assessed using the TapeStation 4150 system (Agilent Technologies). RNA‐seq libraries were prepared using the NEBNext Ultra II Directional RNA Library Prep Kit (New England BioLabs) or TruSeq Stranded mRNA Library Prep Kit (Illumina), as per the respective manufacturers' protocols. Libraries were sequenced on either the NextSeq 550 or NovaSeq 6000 platform (Illumina) using 150‐bp paired‐end reads. Raw sequencing data were analyzed using default settings in the CLC Genomics Workbench (v24.0.2; Qiagen). Gene set enrichment analysis (GSEA) was conducted using the GSEA software (v4.3.2) with the Hallmark gene sets (Broad Institute).

### 2.5 Breast Cancer Tissue Processing and Patient‐Derived Organoid Culture

Breast cancer tissue was obtained from surgical or biopsy specimens collected at Hyogo Medical University Hospital. All procedures were approved by the Institutional Review Board of Hyogo Medical University (no. 3940), and written informed consent was obtained from all participants. The study was conducted in accordance with the tenets of the Declaration of Helsinki.

Fresh tissue samples were minced into fragments (1–3 mm^3^), washed thoroughly with D‐BSA (DMEM containing GlutaMAX Supplement [Gibco, cat. no. 10569010], 0.1% bovine serum albumin [Sigma, cat. no. A6003], and 1% penicillin–streptomycin solution [Nacalai Tesque, cat. no. 26253‐84]), and enzymatically digested using 1 mg/mL collagenase (Gibco, cat. no. 17101015) supplemented with 10 μM Y‐27632 (AbMole Bioscience, cat. no. M1817) on a horizontal shaker at 37°C for 0.5–2 h [23, 24]. The resulting cell suspension was filtered, washed, and centrifuged at 450 *g* for 5 min at 8°C. In samples containing visible erythrocyte contamination, red blood cells were lysed with 1 mL red blood cell lysis buffer (Roche, cat. no. 11814389001) for 2 min at room temperature, followed by dilution with 10 mL D‐BSA and centrifugation. The final pellet was resuspended in ice‐cold Cultrex Reduced Growth Factor Basement Membrane Extract (BME), Type 2 (10 mg/mL; Bio‐Techne, cat. no. 3533‐005‐02).

Aliquots of 100 μL of the BME‐cell suspension were plated as small domes in each well of a prewarmed 12‐well suspension culture plate (Greiner, cat. no. 665102) and incubated at 37°C for 30 min [23] to allow gelation. After solidification, 750 μL of breast cancer organoid culture medium was added per well, and the plates were incubated in a humidified atmosphere at 37°C with 5% CO_2_. The medium was refreshed every 4 days, and organoids were passaged every 1–4 weeks.

### 2.6 Animal Models

All animal experiments were conducted in accordance with institutional guidelines at the Animal Research Facility of Hyogo Medical University, following protocols approved by the Institutional Animal Care and Use Committee (IACUC). The facility is accredited by the Association for Assessment and Accreditation of Laboratory Animal Care (AAALAC) and maintained under pathogen‐free conditions. For the establishment of a patient‐derived xenograft (PDX) breast cancer model, female BALB/c nude mice were obtained from CLEA Japan Inc. (Tokyo, Japan). A total of 8 × 10^5^ breast cancer PDO cells, suspended in 50 μL of a 1:1 mixture of culture medium and BME, were orthotopically implanted into the lower mammary fat pad under direct visualization [26]. Tumor fragments (1–2 mm^3^) were subsequently passaged through serial transplantation. Tumor dimensions were measured twice weekly using calipers, and tumor volume was calculated using the cylindrical formula. The mice were randomly assigned to treatment groups (saline, eribulin [ERI], TCZ, and ERI + TCZ) 31 days post‐implantation. Mice were euthanized when tumor volume exceeded 2500 mm^3^. Kaplan–Meier survival analysis was performed to assess treatment efficacy.

### 2.7 Statistical Analysis

Statistical analyses were conducted using GraphPad Prism software. Comparisons between groups were performed using unpaired, two‐tailed *t*‐tests. For comparisons among multiple groups, one‐way analysis of variance (ANOVA) followed by appropriate post hoc tests was used. Results with *p*‐value < 0.05 were considered statistically significant. All experiments were performed in triplicate and independently repeated at least three times. In vivo experiments were conducted in triplicate, with each group consisting of a minimum of four mice.

## 3 Results

### 3.1 IL‐6 Production in Response to Eribulin Treatment in Breast Cancer Cell Lines Under TNF‐α Stimulation

**FIGURE 1:**
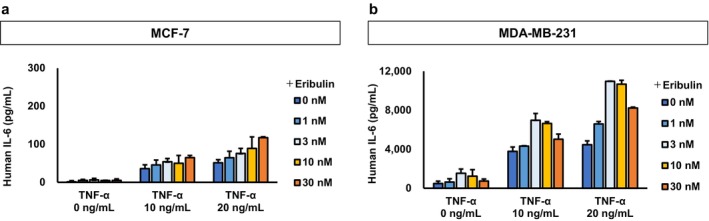
Interleukin‐6 (IL‐6) levels in culture media following tumor necrosis factor alpha (TNF‐α) stimulation in breast cancer cell lines. IL‐6 concentrations were measured in the culture supernatant of MCF‐7 (a) and MDA‐MB‐231 (b) cells, with or without TNF‐α stimulation at the indicated concentrations.

To investigate the relationship between eribulin treatment and IL‐6 production in vitro, we assessed IL‐6 levels in the culture supernatants of breast cancer cells treated with increasing concentrations of eribulin (Figure 1). Baseline IL‐6 expression was higher in MDA‐MB‐231 cells than in MCF‐7 cells. In MCF‐7 cells, eribulin alone did not alter IL‐6 levels in the absence of TNF‐α (Figure 1a). However, stimulation with 10 ng/mL TNF‐α increased baseline IL‐6 levels, which further increased in response to increasing eribulin concentrations. A higher dose of TNF‐α (20 ng/mL) led to a more pronounced baseline increase in IL‐6 levels, with a further dose‐dependent increase upon eribulin treatment. In MDA‐MB‐231 cells, eribulin alone modestly elevated IL‐6 levels (Figure 1b). Stimulation with 10 ng/mL TNF‐α further enhanced IL‐6 production, and this enhancement was potentiated by increasing eribulin concentrations.

**FIGURE 2:**
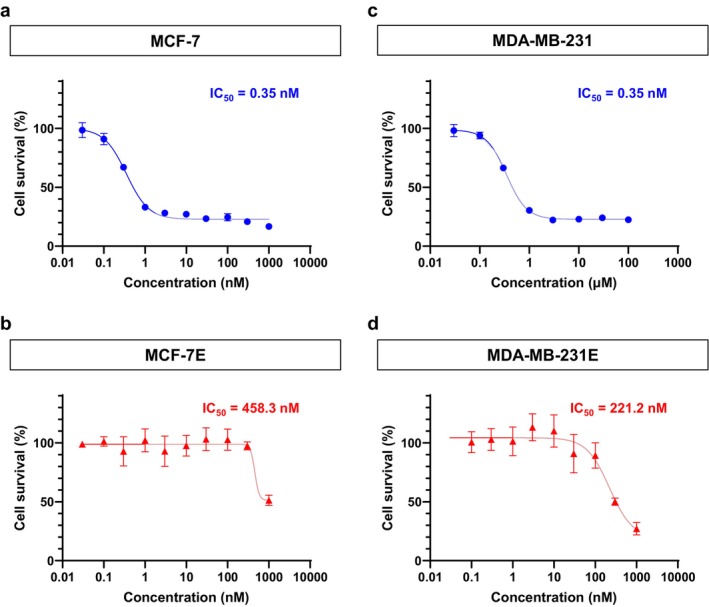
Dose–response curves of eribulin in parental and eribulin‐resistant breast cancer cell lines. (a, b) Dose–response curves and IC_50_ values for eribulin in parental MCF‐7 cells (a) and eribulin‐resistant MCF‐7E cells (b). (c, d) Dose–response curves and IC_50_ values for eribulin in parental MDA‐MB‐231 cells (c) and eribulin‐resistant MDA‐MB‐231E cells (d).

Next, we established eribulin‐resistant cell lines (MCF‐7E and MDA‐MB‐231E) by subjecting parental MCF‐7 and MDA‐MB‐231 cells to long‐term culture with eribulin (Figure 2). Cells cultured with eribulin for 96 h were assayed for sensitivity to eribulin using Cell Count Reagent SF. The half maximal inhibitory concentration (IC_50_) of eribulin for MCF‐7E cells was over 1000‐fold higher than that for the parental MCF‐7 cells (Figure 2a,b). Similarly, the IC_50_ for MDA‐MB‐231E cells was more than 500‐fold higher than that for the parental MDA‐MB‐231 cells (Figure 2c,d), indicating that both cell lines had acquired significant resistance to eribulin.

### 3.2 RNA‐Seq Analysis and Activation of the IL‐6–JAK–STAT Pathway in Eribulin‐Resistant Strains

**FIGURE 3:**
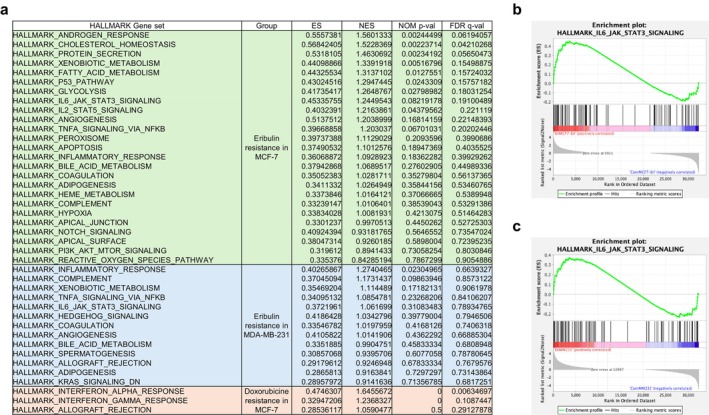
RNA‐seq‐based pathway analysis in breast cancer cell lines. (a) Pathway analysis of eribulin‐resistant MCF‐7E and MDA‐MB‐231E cells and doxorubicin‐resistant MCF‐7D cells. (b, c) Gene set enrichment analysis of the IL‐6–JAK–STAT3 signaling pathway in MCF‐7E (b) and MDA‐MB‐231E (c) cells.

The RNA‐seq analysis revealed significant activation of multiple signaling pathways in the eribulin‐resistant cell lines, including the IL‐6–JAK–STAT pathway, which was not activated in the doxorubicin‐resistant MCF‐7D cells (Figure 3a). The GSEA confirmed the upregulation of IL‐6–JAK–STAT pathway‐related genes in both MCF‐7E and MDA‐MB‐231E cells (Figure 3b,c).

### 3.3 Inhibition of Cell Proliferation by the Combination of Eribulin and TCZ

**FIGURE 4:**
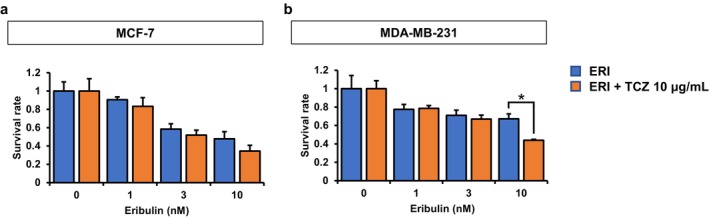
Inhibition of breast cancer cell viability by eribulin (ERI) in combination with tocilizumab (TCZ). (a, b) MCF‐7 (a) and MDA‐MB‐231 (b) cells were treated with ERI at the indicated concentrations in the presence or absence of 10 μg/mL TCZ. *p < 0.05.

MCF‐7 (Figure 4a) and MDA‐MB‐231 (Figure 4b) cells were treated with eribulin in the presence or absence of 10 μg/mL TCZ for 48 h, and impedance‐based cell viability measurements were performed to evaluate the combinatorial effects. As shown in the leftmost two bars of the graph depicting conditions with 0 nM eribulin, TCZ alone did not affect cell viability in either cell line (Figure 4a,b). However, co‐treatment with eribulin resulted in a significant synergistic inhibition of proliferation of MDA‐MB‐231 cells, whereas the effect on MCF‐7 cells was minimal.

### 3.4 Combination Therapy With Eribulin and TCZ Suppresses Tumor Growth In Vivo

**FIGURE 5:**
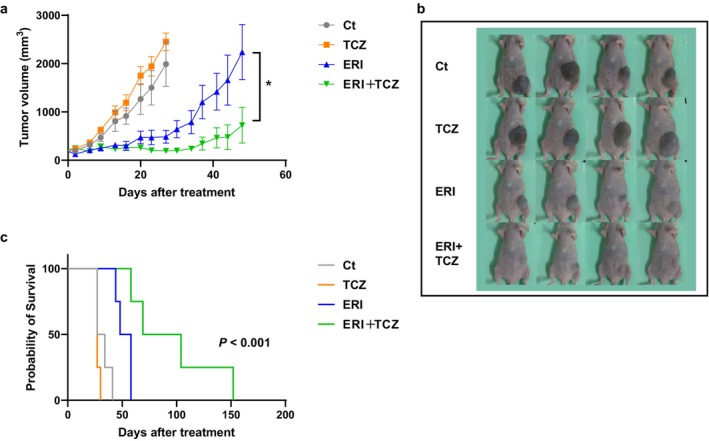
Therapeutic efficacy of eribulin (ERI), tocilizumab (TCZ), or their combination in a patient‐derived xenograft (PDX) model established with a sample from a patient with breast cancer with acquired eribulin resistance. (a) Tumor volume in mice treated with control (Ct), ERI, TCZ, or ERI + TCZ. *p < 0.05. (b) Representative macroscopic images of tumors in the Ct, ERI, TCZ, and ERI + TCZ groups on day 27 of treatment. (c) Kaplan–Meier survival curves for the Ct, ERI, TCZ, and ERI + TCZ groups in the PDX model.

To evaluate the in vivo efficacy of eribulin and TCZ combination therapy, we employed a PDX model generated from eribulin‐resistant breast cancer tissue. Tumor growth was significantly suppressed in the ERI + TCZ group compared with that in the ERI‐only group, as measured by tumor volume (*p* < 0.05, Figure 5a,b). The Kaplan–Meier survival analysis further demonstrated significantly prolonged survival in the ERI + TCZ group (*p* < 0.001, Figure 5c), indicating that IL‐6 receptor inhibition enhances the therapeutic efficacy of eribulin.

## 4 Discussion

Previous clinical studies have demonstrated that peripheral blood biomarkers such as ALC are associated with the therapeutic efficacy of eribulin in patients with advanced breast cancer, suggesting the involvement of an immunological component in its mechanism of action. However, the molecular mediators underpinning this relationship remained unclear until recently [17]. We previously identified serum IL‐6 as a prognostic biomarker in patients with advanced recurrent breast cancer undergoing eribulin therapy, implicating IL‐6 in the development of drug resistance [17]. Nevertheless, the mechanistic contribution of IL‐6 to eribulin resistance and its potential utility as a therapeutic target had not been fully characterized. In this study, we demonstrated that IL‐6 plays a pivotal role in mediating resistance to eribulin. Importantly, we also found that IL‐6 inhibition potentiated the antitumor effects of eribulin in drug‐resistant breast cancer models, highlighting a novel therapeutic strategy to overcome resistance.

Our findings show that combining TCZ, an IL‐6 receptor inhibitor, with eribulin significantly improved therapeutic efficacy in PDX models established from patients with acquired eribulin resistance. IL‐6/IL‐6 receptor blockade using agents such as TCZ has been proposed as a promising therapeutic approach in various malignancies [27]. For instance, in cholangiocarcinoma, IL‐6 inhibition has been shown to counteract gemcitabine resistance by suppressing STAT3 activation driven by IL‐6–producing cancer‐associated fibroblasts [28]. Similarly, in breast cancer, IL‐6–mediated expansion of the cancer stem cell population contributes to trastuzumab resistance, and IL‐6 receptor blockade has been reported to reverse this phenotype [29]. In our study, the pronounced antitumor effect of IL‐6 inhibition observed in vivo suggests that both tumor‐intrinsic and microenvironmental IL‐6 signaling may contribute to eribulin resistance. The enhanced efficacy in the in vivo setting underscores the translational potential of IL‐6 pathway targeting for clinical application.

Our results showed no significant difference in the intrinsic response to eribulin between MCF‐7 and MDA‐MB‐231 cells; however, the synergistic effect of eribulin with TCZ was more pronounced in MDA‐MB‐231. These two cell lines represent different breast cancer subtypes, raising the possibility that eribulin responsiveness may vary by subtype. However, previous clinical studies have not provided consistent evidence for subtype‐specific differences in eribulin efficacy [30, 31], and previous preclinical study reports also suggest no significant difference in eribulin responsiveness between MCF‐7 and MDA‐MB‐231 cells [32]. In our study, the stronger effect of TCZ with eribulin on MDA‐MB‐231 cells, which exhibit higher IL‐6 dependency, suggests that IL‐6 dependency may contribute to eribulin resistance. Whether IL‐6 dependency is intrinsically linked to breast cancer subtype remains uncertain, warranting further investigation.

IL‐6 has also been reported to influence the tumor immune microenvironment by promoting the induction of suppressive immune cells, such as regulatory T cells (Tregs) and MDSCs, thereby facilitating cancer progression [33, 34]. Indeed, our previous study revealed that the proportion of MDSCs significantly increases in patients with high IL‐6 expression [17]. In the present study, inhibition of IL‐6 when combined with eribulin synergistically suppressed tumor progression in a PDX model. Considering the immunodeficient nature of the nude mouse model, which lacks T cells, the observed antitumor effects may be mediated by natural killer (NK) cells or other immune components. Taken together, our results suggest that IL‐6 contributes to eribulin resistance through both direct effects on cancer cells and modulation of the tumor immune microenvironment.

This study has certain limitations. First, the diverse mechanisms by which IL‐6 mediates drug resistance were not fully delineated. Nonetheless, our data provide strong evidence that IL‐6 plays a functional role in eribulin resistance and that IL‐6 pathway inhibition is a promising therapeutic target. It also remains unclear whether IL‐6–mediated resistance is specific to eribulin or represents a more generalizable mechanism applicable to other chemotherapeutic agents. The RNA‐seq–based pathway analysis revealed enrichment of IL‐6–associated signaling in two independently derived eribulin‐resistant cell lines, but not in a doxorubicin‐resistant counterpart, suggesting that IL‐6 pathway activation may be specifically induced by eribulin exposure. In addition, Figures 2 and 4 were generated using different measurement methods and experimental conditions, which may account for the discrepancy in the apparent sensitivity of cells to eribulin. Such methodological differences can influence growth inhibition readouts and should be considered when interpreting the results. Further studies are warranted to elucidate the molecular basis of IL‐6 pathway activation and its role in acquired resistance to eribulin.

In conclusion, this study demonstrates that IL‐6 contributes to eribulin resistance in breast cancer and that targeting IL‐6 may represent a potential therapeutic strategy to overcome this resistance. Further investigations are warranted to elucidate the precise mechanisms by which IL‐6 mediates eribulin resistance and to explore the translational potential of IL‐6 inhibition in clinical settings.

## Author Contributions

**Akira Hattori:** conceptualization, investigation, writing – original draft. **Masayuki Nagahashi:** conceptualization, funding acquisition, investigation, methodology, project administration, writing – original draft. **Miki Komatsu:** methodology, writing – original draft, investigation. **Sayaka Urano:** investigation, writing – original draft. **Mamiko Kuroiwa:** investigation, writing – review and editing. **Yosuke Matsushita:** investigation, methodology, formal analysis, writing – original draft. **Toyomasa Katagiri:** formal analysis, supervision, writing – review and editing. **Masafumi Shimoda:** supervision, writing – review and editing. **Yasuo Miyoshi:** funding acquisition, project administration, supervision, writing – review and editing.

## Ethics Statement

The study was approved by the Institutional Review Board of Hyogo Medical University (approval no. 3940). Written informed consent was obtained from all participants, and all procedures involving human subjects were conducted in accordance with the tenets of the Declaration of Helsinki. Animal experiments were conducted in compliance with institutional guidelines at the Animal Research Facility of Hyogo Medical University. Registry and the registration no. of the study/trial: N/A.

## Conflicts of Interest

M. Nagahashi has received honoraria from Chugai, AstraZeneca, Eli Lilly, Pfizer, Novartis, MSD, Taiho, Daiichi Sankyo, Eisai, Kyowa Kirin, and Denka. M. Shimoda has received honoraria from Chugai, Pfizer, MSD and Eisai. Y. Miyoshi has received research funding and honoraria from Eisai, Chugai, AstraZeneca, Eli Lilly, Pfizer, MSD, Kyowa Kirin, Daiichi Sankyo, and Taiho. A. Hattori, M. Komatsu, S. Urano, M. Kuroiwa, Y. Matsushita, and T. Katagiri declare no conflicts of interest.

## Data Availability

The data that support the findings of this study are available on request from the corresponding author. The data are not publicly available due to privacy or ethical restrictions.
